# The Clubroot Pathogen (*Plasmodiophora brassicae*) Influences Auxin Signaling to Regulate Auxin Homeostasis in Arabidopsis

**DOI:** 10.3390/plants2040726

**Published:** 2013-11-27

**Authors:** Linda Jahn, Stefanie Mucha, Sabine Bergmann, Cornelia Horn, Paul Staswick, Bianka Steffens, Johannes Siemens, Jutta Ludwig-Müller

**Affiliations:** 1 Institut für Botanik, Technische Universität Dresden, 01062 Dresden, Germany; E-Mails: Linda.Jahn@mailbox.tu-dresden.de (L.J.); Mucha.Stefanie@web.de (S.M.); bine.schu@web.de (S.B.); Cornelia.Horn@mailbox.tu-dresden.de (C.H.); Johannes.Siemens@hu-berlin.de (J.S.); 2 Department of Agronomy and Horticulture, University of Nebraska, 379 Keim, Lincoln, NE 68521 USA; E-Mail: pstaswick1@unl.edu; 3 Botanisches Institut, Universität Kiel, Am Botanischen Garten 5, 24118 Kiel, Germany; E-Mail: bsteffens@bot.uni-kiel.de

**Keywords:** ABP1, *Arabidopsis thaliana*, auxin homeostasis, auxin receptors, clubroot disease, GH3 proteins, *Plasmodiophora brassicae*, potassium channel inhibitors, tetraethylammonium, TIR1

## Abstract

The clubroot disease, caused by the obligate biotrophic protist *Plasmodiophora brassicae*, affects cruciferous crops worldwide. It is characterized by root swellings as symptoms, which are dependent on the alteration of auxin and cytokinin metabolism. Here, we describe that two different classes of auxin receptors, the TIR family and the auxin binding protein 1 (ABP1) in *Arabidopsis thaliana* are transcriptionally upregulated upon gall formation. Mutations in the *TIR* family resulted in more susceptible reactions to the root pathogen. As target genes for the different pathways we have investigated the transcriptional regulation of selected transcriptional repressors (*Aux/IAA*) and transcription factors (*ARF*). As the TIR pathway controls auxin homeostasis via the upregulation of some auxin conjugate synthetases (GH3), the expression of selected *GH3* genes was also investigated, showing in most cases upregulation. A double *gh3* mutant showed also slightly higher susceptibility to *P. brassicae* infection, while all tested single mutants did not show any alteration in the clubroot phenotype. As targets for the ABP1-induced cell elongation the effect of potassium channel blockers on clubroot formation was investigated. Treatment with tetraethylammonium (TEA) resulted in less severe clubroot symptoms. This research provides evidence for the involvement of two auxin signaling pathways in Arabidopsis needed for the establishment of the root galls by *P. brassicae*.

## 1. Introduction

The clubroot disease of the Brassicaceae is one of the most damaging diseases within this plant family [1]. The growth of clubroot-infected plants is stunted compared to healthy plants and the root system shows typical gall formation. At maturity, the galls turn brown and soft as the tissue decomposes so that the spores are liberated from the plant tissue. These spores can remain infectious for at least 15 years [1]. This economically important pathogen infects a range of crop plants within the Brassicaceae. In addition, *Arabidopsis thaliana* is a good host, making molecular and functional studies feasible [2]. The disease is still difficult to control by either chemical or cultural means [3].

Obligate biotrophic plant pathogens like *Plasmodiophora brassicae* establish an intricate interaction with their host during at least some parts of the infection process, because of their dependence on host carbon sources. They influence host physiology and alter host regulatory networks over a wide range of its genome. Especially the plant's hormonal balance is altered by this interaction [4]. The changes in host hormone metabolism are connected to the intracellular life style of this protist. 

The infection process of plants by *P. brassicae* consists of two phases: (1) the primary phase, which is restricted to root hairs and (2) the secondary phase, which occurs in the cortex and stele of roots and hypocotyl and leads to abnormal development [5]. Especially during this later phase the host root responds to infection by increased cell division rates followed by hypertrophy of infected cells. These harbor first the plasmodia of *P. brassicae*, probably dividing together with the host cells. Later, the plasmodia grow and the host cell increases concomitantly in size. These enlarged host cells can reach at least ten times the size of uninfected cells [6]. While cell division has been attributed to the action of auxins and cytokinins [7], cell enlargement has been so far linked exclusively to higher auxin concentrations [8] and synthesis [9,10]. The induction of auxin in Arabidopsis correlates with an increase in seedling growth and Xyloglucan-Endo-Transferase/Hydrolase leading to cell elongation [8]. In addition, a microarray [7] revealed that genes involved in cell division and expansion such as cell cycle genes and expansins are upregulated, especially at the first analyzed time point of the disease (10 days after inoculation) [2]. Consequently, altering hormone concentrations has led to reduced clubroot symptoms [7,11]. Despite a long lasting research on hormonal events, the exact signaling and control mechanisms are still not known. Therefore, it was investigated, which auxin signaling pathway(s) contribute to clubroot formation. 

**Figure 1:**
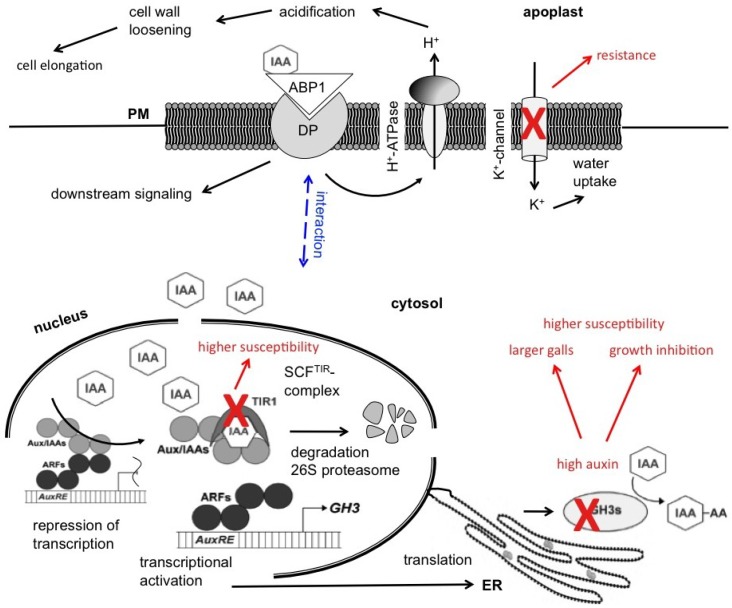
Model for the auxin-dependent degradation of Aux/IAA proteins via the TIR receptor family to induce gene expression by auxin in Arabidopsis and the possible involvement of *P. brassicae* in this process (indicated in red). The role for a second auxin receptor, ABP1, might lie in processes at the plasma membrane, leading in turn to cell elongation.

Auxin signaling is regulated by two types of receptors: the nuclear-localized TIR/AFB family [12,13] and the plasma membrane associated auxin binding protein 1 (ABP1) [14] (Figure 1). The nuclear signaling pathway leads to the activation of the transcriptional response via auxin perception by the F-box protein TIR1 (transport inhibitor response 1). F-box proteins function as substrate recognition modules for the multisubunit complex of ubiquitin ligases (also called Skp1**-**Cullin1**-**F-box protein (SCF); here SCF^TIR^) [15]. The F-box protein TIR1 is the receptor, which recruits, by binding to auxin, the protein target that is designated for degradation by ubiquitination [13,16]. Polyubiquitinated proteins are then transferred into the 26S proteasome and degraded. 

Auxin-mediated transcriptional response is controlled by negative (Aux/IAA) and mainly positive (ARF) regulators [16]. ARFs (auxin response factors) bind to the auxin responsive elements (AuxRe) in respective promoters of auxin-inducible genes for transcriptional activation, but some ARFs are also inhibitors [17]. Both, Aux/IAAs and ARFs are present as large families. The Aux/IAA proteins bind to the ARFs through homologous domains in both proteins and thereby repress auxin-regulated transcription [18]. The Aux/IAA proteins are short-lived, because they are degraded via the SCF^TIR^ pathway and their degradation is promoted by auxin. Many mutations in *Aux/IAA* genes are stabilizing the resulting protein, because degradation domains are affected and thus they constitute gain-of-function mutations [18]. 

Among the targets of ARFs are genes encoding proteins involved in the regulation of auxin homeostasis. The IAA amino acid conjugate synthetases (GH3) have been first recognized as auxin-induced genes [19]. Later it was discovered that it is a large gene family and that some proteins are involved in the conjugation of IAA to various amino acids [20]. GH3 proteins are involved in various responses of plants to abiotic and biotic stresses [21]. Especially the protein GH3.5 seems to play various roles in addition to the synthesis of IAA amino acid conjugates. It also conjugates the plant defense signal salicylic acid to amino acids [22] and is involved in the synthesis of the Arabidopsis phytoalexin camalexin [23]. One family member, GH3.11 (JAR1), catalyzes the formation of the isoleucine conjugate of jasmonic acid (JA) [24]. Contrary to the inactivation of IAA by conjugation, the isoleucine conjugate of JA is active, because it alone binds to the JA receptor COI1 [25]. 

ABP1 has been shown to control events at the plasma membrane by the regulation of membrane fluxes leading in consequence to cell elongation. ABP1 has an ER retention sequence, but it is hypothesized that for action at the plasma membrane some protein “escapes” its intracellular location [14]. In addition, there is no transmembrane sequence present in ABP1, which led to the speculation that a docking protein could be responsible for anchoring ABP1 in the membrane (Figure 1). ABP1 is involved in protoplast swelling [26], which is an indication for its role *in planta* in cell elongation. In addition, many growth processes seem to rely on ABP1 activity, for example an ABP1 knockout mutant is embryo lethal [27]. Downstream of ABP1 the action of ATPases and ion channels was postulated. Both, a H^+^-ATPase and K^+^-channels are needed for the auxin-mediated cell elongation response [14]. K^+^-channels necessary for the osmotic changes occurring during cell elongation can be induced by auxin in maize and Arabidopsis [28]. In contrast, the TIR pathway can be responsible for the increased gene expression for these channels in response to auxin, linking the two pathways. 

Initial evidence for the involvement of the TIR pathway in the clubroot disease came from Alix *et al*. [29] who reported the partial resistance of the Arabidopsis *axr3-1* mutant. The *AXR3* gene encodes the transcriptional repressor IAA17, a member of the Aux/IAA family. The transcript is stabilized in the mutant, so that the transcriptional activation cannot take place. Here, we have investigated the contribution of both auxin signaling pathways mentioned above to the clubroot symptom development. In addition, targets of both pathways were functionally investigated for their roles during disease progression.

## 2. Results and Discussion

### 2.1. The Auxin Signaling Receptors TIR1 and AFB1 Are Transcriptionally Upregulated in Clubroots

The role of plant hormones during the infection of Brassicaceae roots with the obligate biotrophic protist *Plasmodiophora brassicae* has been studied over the decades. The increase in auxin and cytokinin is well-documented and experimental evidence for the biosynthetic pathways leading to these increases have been obtained [4]. The role of auxin transport is less well understood, but there is also experimental evidence that IAA transport plays a role in clubroot formation [10,30]. In contrast, the signal transduction pathways for IAA and their subsequent targets have not well been studied. Only a few experimental data on the involvement of genes from the auxin signaling pathways are available. The *axr3* mutant is more resistant to *P. brassicae*, while for the *tir1* mutant no phenotypical changes after infection with the clubroot pathogen were found [29]. 

As *Arabidopsis thaliana* is a good host for *P. brassicae* we used the ATH1 Affymetrix 22K microarray to investigate host gene expression during the development of the disease on a broader basis [7] and focused on genes involved in the regulation of the auxin pool and auxin-induced gene expression (Tables S1 and S2). The data were also compared to other publicly available microarray datasets to analyze additional features, such as auxin treatments (Table S2) [31]. 

As mentioned, IAA is perceived by a family of F-box proteins called the TIR1 (transport inhibitor response1)/AFB (auxin signaling F-box protein). Phylogenetic studies showed that these proteins fall into four clades in flowering plants [32]. TIR1 and AFB1 show high sequence homology to each other [12], whereas the other AFBs are more distantly related. For the auxin receptors investigated here the binding of IAA was demonstrated [32]. One group of AFBs was found to negatively regulate the auxin response, because loss of AFB4 resulted in growth phenotypes consistent with auxin hypersensitivity [33]. In addition, the AFB4 clade was identified as the major target of auxinic herbicides [33].

**Figure 2:**
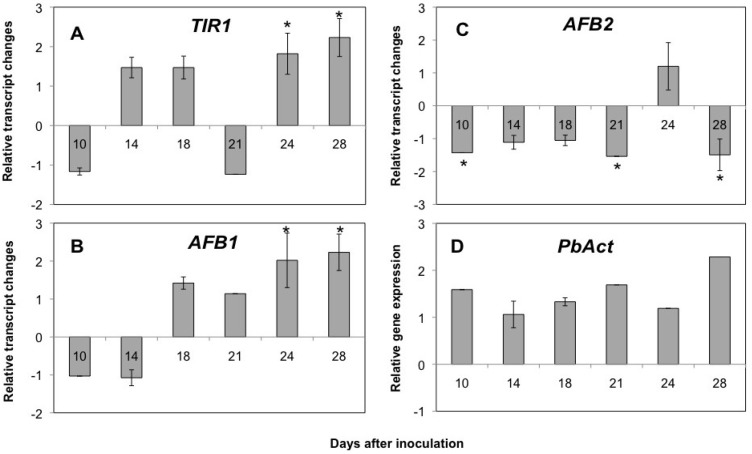
(**A**–**C**) Expression of three representatives of auxin receptors from the TIR family during the clubroot disease. *TIR1*, *AFB1*, and *AFB2* expression was normalized to the gene encoding the mitosis protein *YLS8* (*YELLOW-LEAF-SPECIFIC* *GENE 8*) of Arabidopsis. Values are mean of at least three independent experiments ± SE. Significant differences are indicated by an asterisk (*p* ≤ 0.05). (**D**) Relative expression of the *P. brassicae actin* gene during the development of the root gall.

Our microarray analysis showed an upregulation of AFB1 during clubroot (24 dai), so the expression of the TIR1/AFB1 pair of auxin receptors was analyzed. Using RT-PCR an upregulation for *TIR1* and *AFB1* during later time points (24 and 28 days after inoculation; dai) of the disease development is shown (Figure 2). 

This is a time point when galls are clearly visible and cell division, as well as cell elongation, occur in the infected roots [7]. Cell elongation is connected to the presence of plasmodia containing cells and high auxin concentrations have been hypothesized for this cell type to occur. On the contrary, a downregulation of *AFB2* transcript was found at most time points (Figure 2A). The presence of *P. brassicae* was detected using the relative transcription of *actin* from the pathogen (*PbAct*) (Figure 2B). These data suggest that auxin signaling is dependent on the TIR pathway during the time frame where the major gall development occurs. 

**Figure 3:**
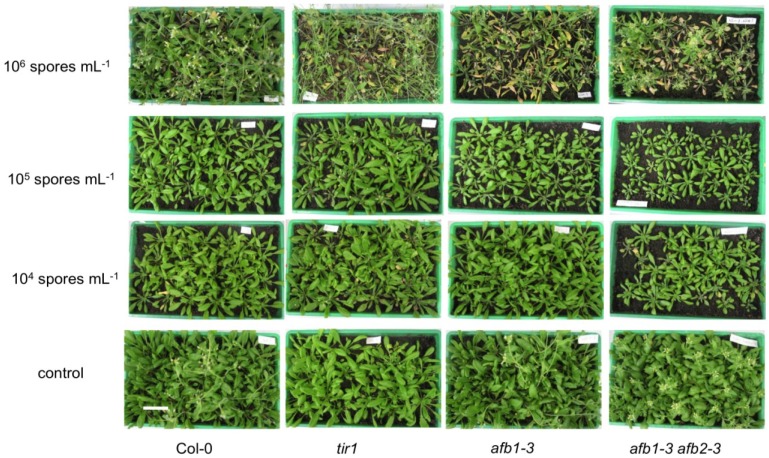
Mutant analysis of *tir1*, *afb1*, and *afb1 afb2* double mutant with respect to their aboveground phenotype 28 days after inoculation with *P. brassicae*. The shoot growth of wild type and three different mutant plants is shown after inoculation with three different spore densities. The experiment was reproduced three times and the results of a typical dataset shown. The bar represents 4 cm and the size is the same for all panels.

**Figure 4:**
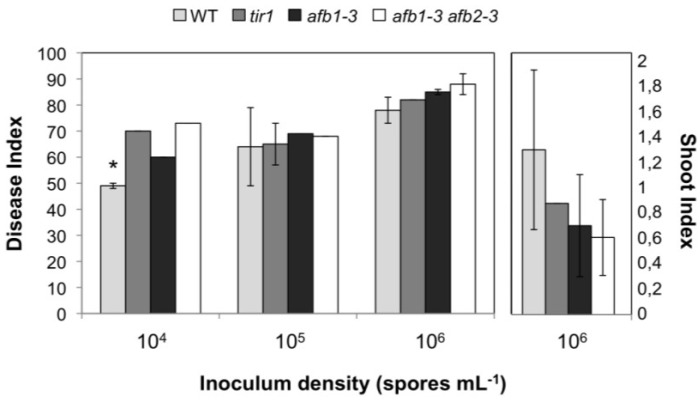
Mutant analysis of *tir1*, *afb1*, and *afb1 afb2* double mutant with respect to their gall development 28 days after inoculation with *P. brassicae*. Disease index (DI) and Shoot index (SI) of *tir1*, *afb1*, and *afb1 afb2* mutants 28 dai. Both values are measures for disease severity. The infection was done with different spore densities. For each experiment at least 60 plants per mutant and inoculation condition were analyzed. Values are means ± SE of three independent experiments. The asterisk indicates a significant difference at α = 0.05, based on Kruskal-Wallis analysis and mean rank comparison.

Single mutants in the *TIR1*, *AFB1*, and a double mutant in the *AFB1* and *AFB2* genes were then tested for phenotypic changes after infection with the clubroot pathogen (Figure 3 and Figure 4). 

High inoculum densities were used to investigate tolerance or resistance phenotypes, because plants, which show a grade of tolerance against *P. brassicae*, should show a low disease index compared to wild type plants with a high index. On the contrary, low inoculum densities can show higher susceptibility. While the disease index here is expected to be low for wild type plants, mutant lines with a higher index are regarded as more susceptible. At different inoculum densities, no differences in aboveground phenotypes of single mutants could be observed compared to the wild type. This is in accordance with observations made by Alix *et al*. [29] for the *tir1* mutant. The phenotypic changes concerning growth patterns are only subtle for single receptor mutants [34]. Creating multiple mutants resulted in stronger growth defect phenotypes depending on the tissue, which resulted in complex phenotypes obtained for different mutant combinations [32,34]. Under the biotic stress conditions investigated here, a double mutant *afb1 afb2* showed alterations in phenotype, *i.e*., it was more susceptible to clubroot (Figure 3), because shoot growth was inhibited already at lower spore densities. 

Differences in growth could be quantified using the shoot index, even though variations were quite high (Figure 4). The disease index (DI) as a parameter for the severity of the disease was similar at higher spore densities (10^5^ and 10^6^ spores mL^−1^) used as inoculum, whereas some differences could be observed at lower spore densities (10^4^ spores mL^−1^), indicating by a higher DI that all mutants were more susceptible than wild type (Figure 4). 

Higher order mutants were not considered for testing in response to clubroot due to their dwarfed phenotype. These data suggest a role for the F-box type auxin receptors for clubroot development. The higher susceptibility of the double mutants can be interpreted as missing downstream gene expression, for example the *GH3* genes, which encode proteins involved in auxin homeostasis (see Section 2.3). Other auxin-induced genes could be involved directly in cell cycle regulation or cell expansion, which is an important factor in club development [2,35]. Finally, potassium channels could be transcriptionally regulated by the TIR signaling pathway (see Section 2.5).

### 2.2. Aux/IAA and ARF Genes Are Differentially Regulated during Clubroot

**Figure 5:**
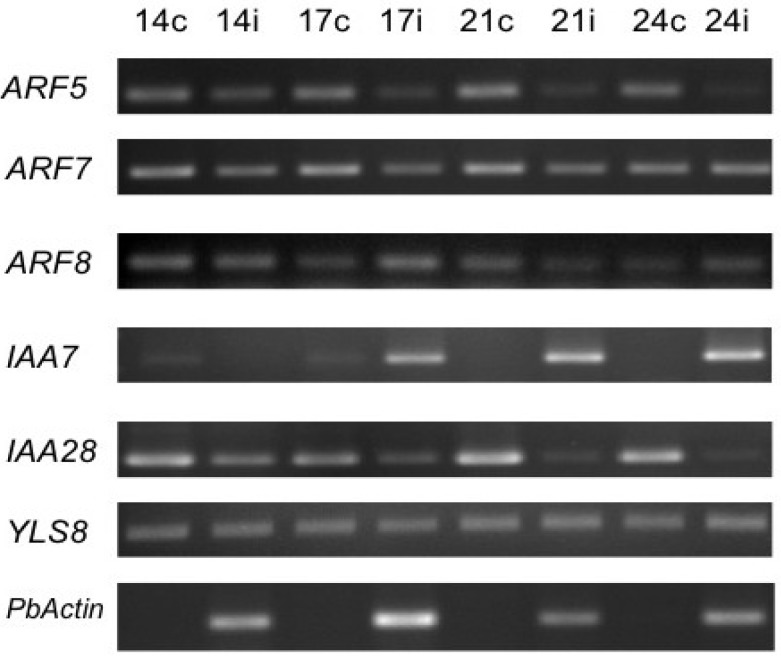
Expression of selected transcriptional repressors (Aux/IAA) and activators (ARF) of the TIR signaling pathway during the clubroot disease. Expression was normalized to the *YLS8* (see Figure 2) of Arabidopsis. The presence of *P. brassicae* is shown by the transcript of the *actin* gene. c = control roots; i = infected roots.

On the basis of microarray results (Table S1) some genes from the transcriptional repressor family Aux/IAA and the transcriptional activators ARFs were chosen for further analysis (Figure 5). *ARF5* encodes the monopteros (MP) protein [36]. Interestingly, triple *tir afb* mutants show a similar embryo phenotype as the *mp* mutant [37]. ARF7 is a positive regulator of lateral root formation [38]. Also, ARF5 and ARF7 partially overlap in their function [36]. *ARF5* and *ARF7* gene expression showed downregulation in infected roots (Figure 5), confirming the results from the microarray (Table S1) [7]. Downregulation of a positive regulator of lateral root formation ARF7 could be interpreted as disturbance of the ordered tissue layers of roots and a reduction of lateral roots in favor of undifferentiated gall formation. In accordance with this hypothesis, it was shown that a cell division reporter, CYCB1;1 was activated in patches after *P. brassicae* infection in roots not yet showing galls, whereas in controls the marker was confined to the root meristems of main and lateral roots [35]. Repression of ARF5 might similarly result in undifferentiated, instead of organized tissue layers, depending on the target genes regulated by this ARF also via different players. ARF5 is involved in the regulation of embryonic roots [39], thus, it might play a role in suppression of root formation in galls.

*ARF8*, in accordance with the microarray data, showed a slight transcriptional upregulation at some time points during disease progression (Figure 5). ARF8 was shown to positively regulate *GH3.5* [40], a member of the auxin conjugate synthetases (see Section 2.3). Also *GH3.5* transcripts were upregulated at some time points (Figure 7B). GH3.5 belongs to the family members, which conjugate IAA to amino acids [20], but also conjugates salicylic acid (SA) with amino acids [22] and it is involved in the synthesis of the Arabidopsis phytoalexin camalexin [23]. In addition, the expression of three other *GH3* genes (*GH3.3*, *GH3.6*, *GH3.17*) was decreased in *arf8* mutants, whereas in *ARF8* overexpressors the same genes showed an increased expression [41]. ARF8 might therefore be an important regulator for the concentration of several signaling molecules in plant defense reactions in addition to IAA concentrations.

Transcriptional responses of two *Aux/IAA* genes were also determined. The gene expression was normalized on the reference gene *YLS8* from the host and *P. brassicae* was determined by relative expression of its *actin* gene. *IAA7* was upregulated in transcription in infected roots, also confirming the microarray data. Transcripts of *IAA28* were always higher in control roots compared to infected roots (Figure 5), again indicating good correlation between RT-PCR data and microarray results. Interestingly, another Aux/IAA gene, *IAA2*, was shown to be upregulated using a *promoter::GUS* fusion in previous work [10], whereas the microarray points to strong downregulation. 

IAA28 is also associated with the (negative) regulation of lateral root formation [42]. As with lateral root formation, the hyperplasia observed in root galls starts from the pericycle [43]. Therefore, the differential regulation of genes in cluboots, which are also involved in the regulation of lateral root development could point to a similar role in root development and club formation. In a healthy plant cell-specific auxin accumulation patterns in xylem pole cells lead to the degradation of the IAA28 repressor protein and determination of precursor cells for lateral root initiation [42]. In clubroots the downregulation of *IAA28* might be a prerequisite for re-embryonalization of the pericycle tissue prior to increased cell division rates or gall development is initially started by massive lateral root development. For IAA7 it has been shown that it can induce growth processes by inhibiting the activity of repressing ARFs [44]. This could explain the upregulation of a transcriptional repressor in growing root galls in response to auxin. Other ARFs, which have not been the subject of investigation here, for example ARF17, are also known to control *GH3* transcriptional response, especially that of *GH3.2* and *GH3.3*, but not *GH3.5* [45]. 

Under sub-threshold auxin concentrations the Aux/IAA proteins heterodimerize with the ARF transcription factors, thereby repressing auxin-inducible gene expression. *P. brassicae* could work under low auxin conditions in repressing Aux/IAA gene function and thus inducing ARF-dependent gene expression of auxin-inducible genes. Under high auxin conditions, auxin binding to the TIR/AFB receptors promotes the recruitment of Aux/IAA proteins to the SCF complexes. Subsequent Aux/IAA ubiquitinylation and proteasome-mediated degradation results in a decline in Aux/IAA proteins, thus de-repressing auxin-inducible gene expression. Therefore, *P. brassicae* could act by up-regulation of the endogenous IAA concentration and use the cellular proteasome machinery to have Aux/IAA proteins degraded so that the transcriptional response could take place. 

**Figure 6:**
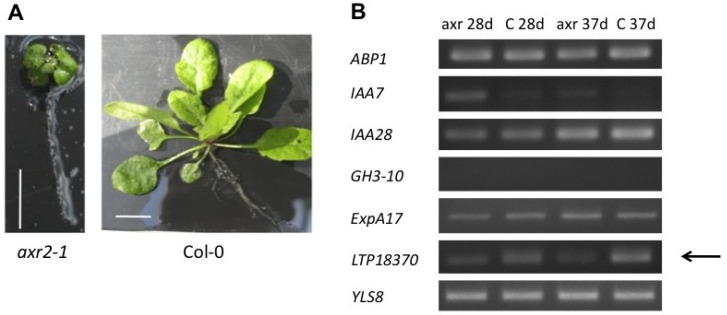
(**A**) Phenotype of *axr2-1* and Col wild type plants. The bar represents 1 cm. (**B**) Using the constitutive repressor mutant *axr2-1* (*iaa7*) to identify putative target genes of the pathway regulated by IAA7 at 28 and 37 days after germination (d). Expression was normalized to *YLS8* of Arabidopsis. The arrow points to a putative target gene, which shows the expected regulation pattern. ABP1: auxin binding protein 1; ExpA17: expansin A17; LTP: lipid transfer protein.

To find putative downstream target genes of this pathway, the gain-of-function mutant *axr2-1* (*iaa7*) was used (Figure 6A). This is not an overexpressor line, but the gain-of-function comes from the stabilization of the transcript encoding the repressor. We reasoned that gene expression altered in this mutant should lead to good candidate genes for further evaluation during the clubroot disease, because of the upregulation of *IAA7* during gall development. As a basis, the microarray data from Siemens *et al*. [7] and Nakamura *et al*. [46] on the *axr2* mutant were used to test this hypothesis (Figure 6B). It was confirmed that *IAA7* expression was higher in the *axr2* mutant compared to the wild type. From other candidate genes chosen the expression of a lipid transfer protein (LTP) displayed the expression pattern expected (upregulation), if the gene would be transcriptionally regulated by this particular Aux/IAA gene (Figure 6B). Indeed, expression analysis showed that this LTP gene was downregulated during clubroot disease development [47]. Furthermore, overexpression of this *LTP* gene in Arabidopsis led to a lower disease index and higher shoot index, both an indication for reduced susceptibility to the clubroot pathogen [47]. The result is in accordance with a role for LTPs in disease development. This could therefore be a promising approach to further identify target genes involved in gall formation, also for other transcriptional regulators of this auxin-signaling pathway.

### 2.3. Several Members of the GH3 Family Are Differentially Regulated during Clubroot Formation

**Figure 7:**
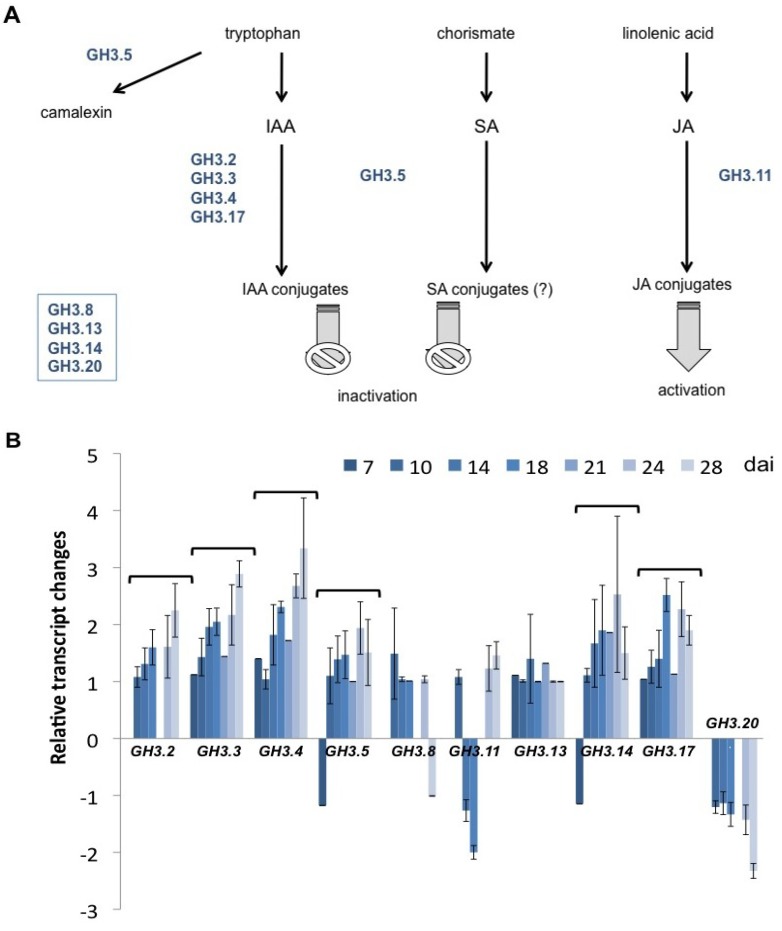
(**A**) Reactions from Tryptophan, IAA, JA and SA to their respective metabolites catalyzed by different GH3 proteins. Only those GH3 genes investigated in this study are shown. Those genes in the box do not convert any of the plant hormones to conjugates. (**B**) Transcriptional regulation of selected *GH3* genes, some of them encoding auxin amino acid conjugate synthetases. Expression was normalized to *YLS8* (see Figure 2) of Arabidopsis. Values are means of at least three independent experiments ± SE. The brackets above a dataset indicate significantly differential regulation at most time points.

Among the possible target of the ARF transcription factors are *GH3* genes [40]. Therefore, we have investigated the transcriptional regulation of selected *GH3* genes. These were chosen according to the results in the microarray analysis (Table S2). GH3.5 was chosen because of its multiple roles in IAA and SA conjugation and also camalexin biosynthesis (Figure 7A). The root tissue specific expression patterns of the selected *GH3* genes are shown in Figure S1 [48]. 

The regulation of auxin concentrations during the development of plants is of importance, because IAA in low concentrations stimulates growth and development, whereas higher concentrations can be toxic to the plant [49]. Therefore, tight control of IAA concentration is necessary for proper plant development. If this homeostasis is disrupted, as in clubroot formation, the proper development of tissues cannot occur. Plants contain low amounts of IAA as the free acid, the active form, and most of their IAA in conjugated forms [50]. These conjugates are thought to be involved in (a) transport of IAA within the plant; (b) the storage and subsequent reuse of IAA; (c) protection of IAA from enzymatic destruction; (d) components of a homeostatic mechanism for control of IAA concentrations; and (e) as an entry route into the subsequent catabolism of IAA [51]. Two main types of conjugated molecules exist: the amide-linked IAA forms bound to one or more amino acids and the ester-linked forms primarily bound to sugar(s). 

The ILR1-like IAA amidohydrolase gene family is involved in the regulation of free IAA concentrations. While for Arabidopsis no differential regulation for this gene family during clubroot has been found, the genes encoding for proteins involved in the conjugation of IAA to amino acids and thereby inactivating the free auxin are strongly upregulated in root galls after *P. brassicae* infection. The results showed that especially the members *GH3.2*, *GH3.3*, *GH3.4*, *GH3.5*, *GH3.14*, and *GH3.17* were upregulated at most time points investigated (Figure 7B). The genes for GH3.8, GH3.13, and GH3.20 were expressed in roots at very low concentrations.

The conjugation of IAA to amino acids by GH3.2, GH3.3, GH3.4, and GH3.17 is most likely a detoxification reaction initiated by the host plant against the high concentrations of auxin generated in the root galls. This assumption is supported by the induction of the *GH3* genes most highly expressed in clubroots also by auxin (Table S2). However, GH3.5 was shown to have additional functions to the IAA conjugate synthetase activity. The defense signal, SA, can be converted to amino acid conjugates [22], which is regarded as inactivation. The upregulation of *GH3.5* in clubroots might also be interpreted as downregulation of plant defense responses via SA. GH3.5 is also involved in camalexin synthesis [23]. However, it should be noted that camalexin does not play a role in the plant's defense against *P. brassicae* [52]. Even though the concentrations of camalexin increased in infected Arabidopsis roots compared to controls, a mutant devoid of camalexin was not more susceptible to *P. brassicae* infection.

*GH3.11* (*JAR1*) and *GH3.20* showed downregulation, whereas *GH3.13* did not show any regulation. *GH3.10* did not show any expression at all in roots (Figure 6B) and was therefore not further investigated. It should be noted that in the case of conjugate formation of JA with the amino acid isoleucine [24] an activation is achieved, *i.e*., the JA-isoleucine conjugate is recognized by the COI1 receptor, which in turn leads, in analogy to auxin, to a degradation of the transcriptional repressor family JAZ [24]. The downregulation of *GH3.11* is in agreement with the assumption that *P. brassicae* downregulates certain aspects of plant defense mechanisms [2]. The *gh3.11* mutant (*jar1*) was shown to be more susceptible to clubroot infection [53]. As a function for GH3.20 has not been described yet, there is no possibility to speculate on the role of transcriptional downregulation during clubroot. Also, *GH3.20* might be truncated [54]. One indication for a role in the auxin-cytokinin interaction came from Jones and Ljung [55]. They analyzed genes involved in auxin metabolism differentially expressed in response to altered cytokinin concentrations and/or responsiveness in Arabidopsis using Genevestigator and found several members of the *GH3* family (*GH3.3*, *GH3.7*, *GH3.8*, *GH3.18*, and *GH3.20*) differentially regulated. These genes are thought to be involved in the feedback metabolic control that regulates relative concentrations of auxin and cytokinin in plants [55]. Both hormones play a role in gall development.

**Figure 8:**
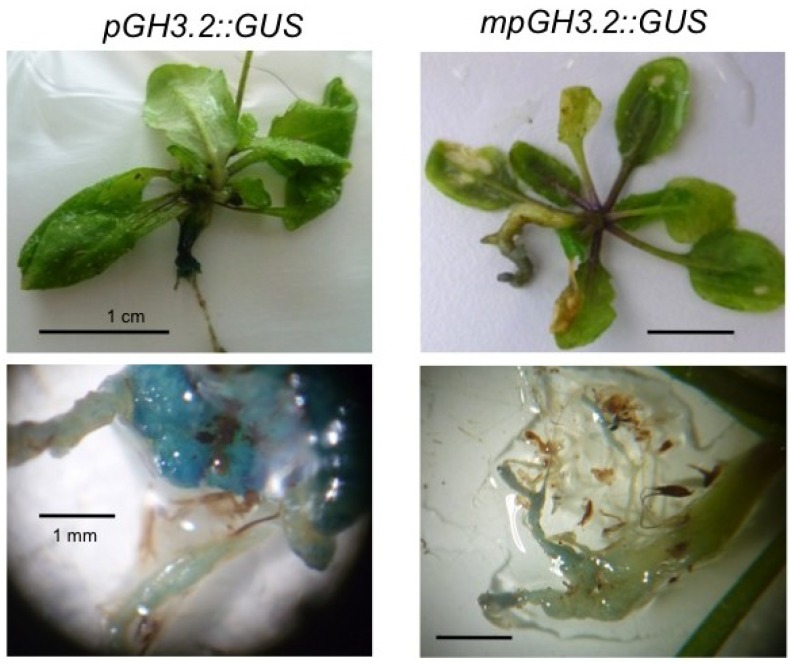
The activation of *GH3* genes by *P. brassicae* occurs most likely via the auxin responsive element in the promoter. On the left side the wild type *GH3.2* promoter was fused to the *GUS* gene (*pGH3.2::GUS*) and on the right side a mutated version of *GH3.2* promoter in the auxin response element (*mpGH3.2::GUS*) was tested. The two upper panels show typical pictures of the comparison of the staining of a mature root gall. The bar represents 1 cm. The two lower panels show parts of the roots with magnification. The bar represents 1 mm.

ARFs interact with Aux/IAA proteins of transcriptional repressors and bind to auxin response elements (AuxRE) in auxin-inducible promoters [19]. Once the repressors are degraded via ubiquitination in the 26S proteasome, the ARFs can induce auxin-responsive genes such as the *GH3*s (Figure 1). To confirm that the transcriptional regulation is via the auxin-inducible gene expression, most likely then via ARFs, we have compared *GH3.2 promoter::GUS* lines with an intact auxin response element (AuxRe) in the promoter and one promoter with a mutated AuxRe (Figure 8) [56]. Indeed, the strong coloring in wild type promoter plants (*pGH3.2::GUS*) was almost abolished in those transgenic lines harboring a mutated *GH3.2* promoter (*mpGH3.2::GUS*). This indicates that also during clubroot the AuxRe elements in the respective promoters need to be intact and that the regulation of the auxin response is via this transcriptional activation in root galls. The inducibility of the wild type promoter by IAA was confirmed, whereas this stimulation was absent in the mutated form (Table S3). 

**Figure 9:**
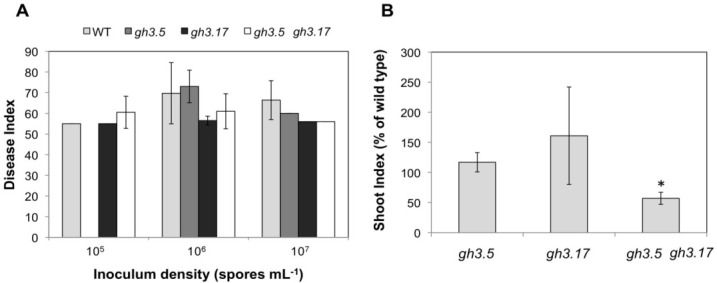
(**A**) Mutant analysis of two *GH3* genes, *gh3.5* and *gh3.17*, as well as the double mutant *gh3.5 gh3.17* for changes in clubroot development 28 dai. Values of the DI at different inoculum densities are means of two to three independent experiments ± SE. (**B**) The same set of plants was evaluated for the shoot index (SI). The values are given as % of wild type. Values of the SI at different inoculum densities are means of three independent experiments ± SE. The asterisk indicates a significant difference at α = 0.05 based on Kruskal-Wallis analysis and mean rank comparison (60 plants per experiment were evaluated).

The analysis of several single knockout mutants in selected *GH3* genes *gh3.3*, *gh3.4*, and *gh3.13* (data not shown), as well as *gh3.5* and *gh3.17* (Figure 9A,B) did not show any significant alterations in the disease phenotype over all different spore concentrations tested.

The mutant *jar1* was previously shown to be more susceptible to the clubroot pathogen [53]. A double mutant of *gh3.5 gh3.17* was more susceptible as indicated for the shoot growth (Figure 9B), whereas the DI did not show any changes compared to wild type (Figure 9A). It should be noted that subsequent work has shown that the *gh3.5* mutant used here only partially reduces transcript concentrations due to a T-DNA insertion in the promoter region [57]. A gene knockout of *GH3.5*, such as *wes1*, might yield greater susceptibility [21]. Other GH3 proteins capable of conjugating IAA to amino acids could substitute for the loss of GH3.5 and GH3.17, explaining the rather weak root phenotype. Therefore, in the future higher order mutants should be included in this research. 

A higher susceptibility could be due to higher auxin concentrations when the conjugation of IAA to the inactive forms is reduced in the double mutant. This in turn can result in increased auxin accumulation, leading first to larger cells and second to growth inhibition of the shoot, if auxin is too high. For example, a double GH3 mutant of the moss *Physcomitrella patens* was reduced in growth, especially under conditions with high auxin concentrations [58]. 

Recently evidence was presented that the IAA amino acid conjugate with aspartate (IAA-Asp) can promote disease progression after bacterial infection in Arabidopsis [59]. IAA-Asp was able to regulate virulence gene expression in the bacterial pathogen, indicating a novel mechanism in adaptation to auxin conjugate formation [59]. These results could also explain the upregulation of several *GH3* genes (Figure 7B), but the results on the *gh3* mutant analysis are not in agreement with such a function (Figure 9). If high *GH3* transcript concentrations would result in disease susceptibility, then a mutation should result in tolerance or resistance to the pathogen, but here our results indicate that the double mutant *gh3.5 gh3.17* was more susceptible than wild type to the protist, at least according to the shoot weight. This is more in accordance with high auxin concentrations.

### 2.4. The Plasma Membrane Associated Receptor ABP1 Is also Upregulated during Clubroot Formation

In addition to the nuclear auxin signaling, there is evidence for a plasma membrane associated auxin receptor, the auxin binding protein 1 (ABP1). Even though this receptor has been known for a very long time, its function is still a matter of debate [60]. A T-DNA insertion mutant indicated a function of the single copy gene ABP1 in embryogenesis because the mutant was embryo-lethal [27]. Since then several conditional ABP1 mutants methods have been generated by biotechnological methods, which demonstrate a role in cell division and elongation [61]. 

**Figure 10:**
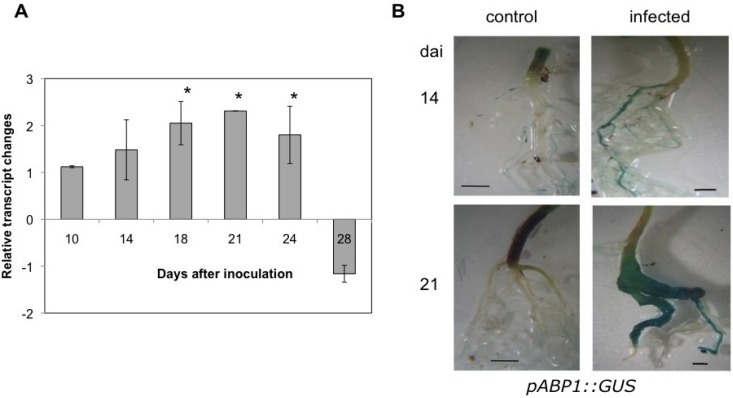
(**A**) Gene expression of auxin binding protein 1 (*ABP1*) during clubroot. Transcript analysis was normalized to *YLS8* of Arabidopsis. Values are mean of three independent experiments ± SE. Significant changes are indicated by an asterisk. (**B**) Promoter::GUS analysis (*pABP1::GUS*) at two time points after inoculation (dai). The bar represents 0.1 cm.

The transcriptional upregulation of the gene encoding the plasma membrane auxin receptor ABP1 starts earlier than the upregulation of the *TIR1* family (Figure 10). From 18 dai the transcripts of *ABP1* are significantly increased in infected roots compared to controls. This time point is usually the time frame when the galls first become visible. The increase is detectable until 24 dai (Figure 10A). 

Klode *et al*. [62] provided tools to investigate the tissue specific localization of ABP1, which were also employed in this study. *Promoter::GUS* lines of Arabidopsis (*pABP1::GUS*) showed clear differences in localization and staining intensities between control and infected roots systems (Figure 10B). In infected roots 14 dai a strong GUS activity is largely visible in the main roots, whereas in control roots of corresponding age the staining is confined to root tips especially of the lateral root system. At 21 dai the staining in the control roots is almost zero, whereas the gall is highly stained. The promoter was not activated by different IAA concentrations (data not shown). The importance of this signaling pathway has to be further demonstrated, e.g., by using conditional *ABP1* mutants.

### 2.5. Treatment with Potassium Channel Inhibitors Increase Tolerance towards the Clubroot Pathogen

The upregulation of *ABP1* warrants the investigation of the role of putative target molecules during gall formation. As ABP1 is reported to be involved in the regulation of cell expansion [14] possible targets are either the H^+^-ATPase at the plasma membrane or the influx potassium channels at the same location (Figure 1). The activity of potassium channels is directly connected with the increase of the turgor pressure within the cell by causing uptake of H_2_O into the vacuole and thus the necessary counterpart to cell wall loosening. K^+^-channel activity can be activated by auxin [63], so the high auxin concentrations during clubroot might directly act on cell elongation via increase of potassium ion influx and subsequent increase in turgor pressure. In addition, K^+^-channel gene transcription can be influenced by auxin and follows the auxin redistribution during gravitropic curvature in maize [64,65]. A role for K^+^-channels in auxin induced cell elongation was also demonstrated in Arabidopsis hypocotyls [66]. After the application of auxin differences between protoplasts from wild type and K^+^-channel mutant *kat1* were monitored. The amplitude of K^+^ in currents in the mutant was reduced two-fold in comparison to wild type, indicating a function for KAT1 in auxin induced potassium influx.

**Figure 11:**
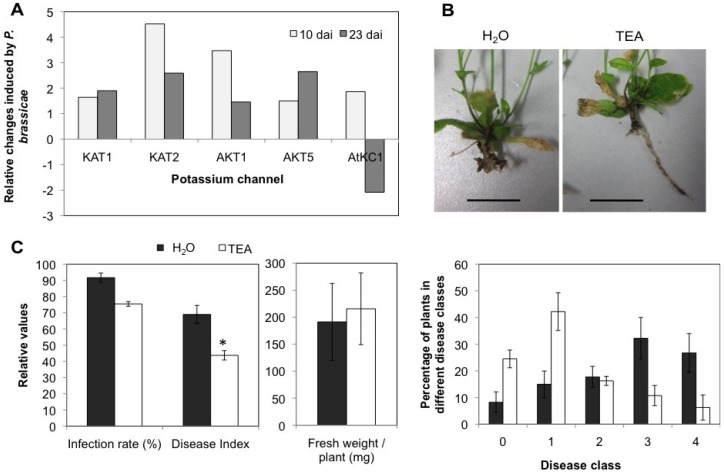
(**A**) Regulation of potassium channels during clubroot according to microarray analysis [7]. All belong to cyclic nucleotide binding/inward rectifier potassium channel. From the K^+^-channels only those have been looked at which show expression in the hypocotyl [28]. (**B**) Phenotype of *P. brassicae*-infected roots 28 dai after treatment with 10 mM TEA 1× a week. The bars represent 1 cm. The phenotype of shoots after the treatment is shown in Figure S1. (**C**) Phytopathological analysis after treatment with 10 mM tetraethylammonium (TEA) a potassium channel blocker showed a reduction of root galls 28 dai. The fresh weight of plants was not altered. Both treatments with TEA (2× and 1×) showed the same results (1× is shown). Data are means of three different experiments ± SE. The asterisk for DI indicates a significant differences at α = 0.05 based on Kruskal-Wallis analysis and mean rank comparison.

According to microarray analyses [7] several inward directed potassium channel genes highly expressed in the hypocotyls of Arabidopsis [28] are upregulated after infection with *P. brassicae* (Figure 11A). All channels belong to the cyclic nucleotide binding/inward rectifier potassium channels. Claussen *et al*. [67] demonstrated that auxin-induced cell elongation could be very effectively inhibited by potassium channel blockers. Therefore, we used the K^+^-channel inhibitor tetraethylammonium (TEA) in treatment of roots starting together with the inoculation time point. The root system was apparently healthier after TEA treatment with longer main roots present compared to H_2_O treatment (Figure 11B). Both, infection rate and DI were significantly reduced after 1× (Figure 11C) treatment with 10 mM TEA. When the plants were treated two times with the same concentration of TEA the results did not differ from the 1× treatment concerning the phenotype of the plants and their response to the clubroot pathogen (data not shown). 

Especially after TEA treatment more plants were found in disease classes 0 (no infection) and 1 (very low disease severity), whereas in controls most plants were categorized to classes 3 and 4 (high disease severity) (Figure 11C). However, it should be noted that the infection rate was also lower after TEA treatment. Thus, it cannot be ruled out that TEA might have a direct effect of *P. brassicae* zoospores, which in turn would lead to lower disease indices. This assumption should be investigated in future work. Even though the aboveground phenotype was altered after TEA treatment, the overall fresh weight of TEA and H_2_O treated the plants was the same (Figure 11C). The aboveground phenotype of TEA treated plants was shorter, but had more branches (Figure S2). This clearly shows that blocking potassium influx renders the Arabidopsis plants more tolerant to clubroot. It also demonstrates that one player in the auxin induced cell elongation in root galls might be K^+^-channels. Since we cannot completely rule out that TEA has a different effect on the clubroot development, it is suggested to use K^+^-channel mutants to further substantiate the claims made here.

## 3. Experimental Section

### 3.1. Plant and Pathogen Material

The ecotype Col-0 of *Arabidopsis thaliana* and the mutant lines for *axr2-1*, *gh3.3*, *gh3.4*, *gh3.5*, *gh3.13*, and *gh3.17* were originally obtained from Nottingham Arabidopsis Stock Centre (NASC). Mutant lines *tir1*, *afb1*, and *afb1,afb2* were provided by Mark Estelle (UC San Diego, La Jolla, CA, USA) and described in [32]. Homozygous mutant lines were tested for transcript reduction by using the PCR conditions and primers described below and in Table S4. The *gh3.5* and *gh3.17* T-DNA insertion mutants used for generating the double mutant were previously described [20]. The double mutant was identified by genotyping individuals from a segregating population after crossing, using the following primers; Lba1 for the T-DNA border, CGGAAAGAGAGAAAA and CGATCCTGTTGATCTCAGGC for *gh3.5*, and TTCAACATCCTTCAAGCCTC and CGAAAAAGAGAGGGAGACAAAG for *gh3.17*. 

The lines carrying the GUS gene under the control of the GH3.2 promoter (*pGH3.2::GUS*, *mpGH3.2::GUS*) were provided by Claus Schwechheimer (TU München, Germany) and were described in [56]. The line carrying the GUS gene under the control of the ABP1 promoter was described in [62].

The *P. brassicae* isolate e3 used in this study was described by Fähling *et al*. [68].

### 3.2. Infection Procedure and Phytopathological Analyses

*Arabidopsis thaliana* wild type Columbia and mutant seedlings were grown in a controlled environment (23 °C, 16 h light, 100 µM photons s^−1^m^−2^) using a compost-sand (9:1, v/v) mixture (pH 5.8). Fourteen-day-old Arabidopsis seedlings were inoculated by injecting the soil around each plant with 2 mL of a resting spore suspension of the pathogen with the spore concentration indicated in Results. The spore suspension was obtained by homogenizing mature clubroot galls of Chinese cabbage (*Brassica rapa*), followed by filtering the spores through gauze (25 µm pore width) and two centrifugation steps (2,500 g, 10 min). The resting spores were resuspended in 50 mM KH_2_PO_4_ buffer (pH 5.8). Disease symptoms were assessed at 28 dai. For the determination of the shoot fresh weight the plants were cut at the top of the hypocotyl. Shoot fresh weight of infected and controls was determined and the shoot index (Si/Sni) [53] was calculated in some experiments. At least 60 Arabidopsis plants were analysed for each line and treatment and the experiments were repeated at least two times. The disease was assessed qualitatively on the basis of the disease index (DI) [53]. The percentage of plants in different disease classes is also specified (0, no symptoms; 1 + 2, roots with light symptoms; 3 + 4, roots with severe symptoms). The qualitative disease assessment data were analysed first using the Kruskal-Wallis test and subsequently by comparing the mean rank differences as described by Siemens *et al*. [53]. Controls were the same age and were treated with 50 mM KH_2_PO_4_ buffer (pH 5.8) instead of spore suspension.

### 3.3. Treatment with Tetraethylammonium

Plants were treated with 10 mM tetraethylammonium (TEA) by pipetting 1× or 2× a week 2 mL of the solution around each seedling on the soil, beginning with the time point of inoculation with *P. brassicae* resting spores. This treatment should ensure that the effect of TEA is mainly excerted on the roots, and the shoots are influenced as little as possible. Controls were watered in the same time intervals with the same volume of H_2_O. 

### 3.4. RNA Extraction and Semiquantitative RT-PCR

Total RNA was isolated from control and infected roots of Arabidopsis at different times after infection using TRIzol^®^ reagent (Invitrogen, Karlsruhe, Germany) according to the manufacturers instructions. To minimize contamination with genomic DNA, RNA was digested with RNase-free DNase (1 U μL^−1^) (Stratagene). First-strand cDNA was prepared from total RNA using M-MLV reverse transcriptase (Invitrogen). 

The primers, annealing temperatures (°C) and times as well as cycle numbers used are listed in Table S4. These sequences are perfect match primers corresponding to a nonhomologous region in other family member genes, either in the coding region or in the 5'-untranslated region (5'-UTR) and coding region. To rule out the amplification of genomic DNA, the primers were chosen so that they spanned an intron in the genomic sequence. Consequently, the resulting PCR amplification product would be larger (data not shown). As reference, all cDNA samples were amplified with *A. thaliana* *YLS8* primers and with *P. brassicae* actin primers to confirm the presence of the pathogen. PCR was performed according to standard procedures using the following programme for amplification: Initial denaturation at 95 °C for 5 min, followed by the number of cycles given in Table S4 of 95 °C/60 s–x °C/x s – 72 °C/60 s (x stands for the conditions given in Table S4), and final elongation at 72 °C for 5 min.

### 3.5. ß-Glucuronidase Staining

The pattern of ß-glucuronidase activity in the promoter::GUS lines was determined in roots by histochemical staining with 5-bromo-4-chloro-3-indolyl glucuronide (X-Gluc) [69]. Plants were incubated in 0.1 M NaH_2_PO_4_/Na_2_HPO_4_ buffer, pH 7.4, containing 10 mM Na_2_EDTA, 0.5 mM K_3_(Fe(CN)_6_), 0.5 mM K_4_(Fe(CN)_6_), 0.5% (w/v) Triton X-100, and 50 µM substrate (X-Gluc dissolved in DMSO). After 1 h incubation at 37 °C, plants were rinsed and placed in 100% acetone for 30 min. After rinsing, the plants were transferred to the NaPO_3_ buffer (pH 7.4) overnight, without the substrate, to block the reaction. 

### 3.6. Re-Analysis of Available Microarray Experiments

The AGI numbers of transcripts for selected genes are from the TAIR database [70]. Transcript concentrations were compared for control and IAA treatment by using the Arabidopsis eFP browser [31,48] and for control and *P. brassicae*-infected roots using the microarray experiment E-MEXP-254 [7]. To compare the *axr2* mutant with wild type data from [46] were used.

## 4. Conclusions

Here, we have shown that the auxin response in clubroot galls induced by *P. brassicae* is mediated by the activation of two signaling pathways, one activating transcriptional responses, controlling among others auxin homeostasis, the other probably directly controlling the events at the plasma membrane leading to cell elongation (see model in Figure 1). Among the players most likely involved in the second are potassium influx channels, which could regulate the turgor pressure within the cell in response to auxin leading to cell elongation. 

The transcriptional response pathway controls a number of auxin-related genes, including the *GH3s*, in response to high auxin concentrations found in clubroot-infected tissue. The GH3 proteins are active in the control of auxin homeostasis leading to the conjugation of free IAA to amino acids and thereby inactivating the auxin molecule. Consequently, a transcriptional regulator of *GH3* gene expression, ARF8, was also upregulated in root galls. Mutant analysis with *afb* receptor mutants as well as *gh3* mutants corroborated the involvement of these auxin pathways in the clubroot disease.

## Supplementary Files

Supplementary File 1

## References

[1] Dixon G.R. (2009). The occurrence and economic impact of *Plasmodiophora brassicae* and clubroot disease. J. Plant Growth Regul..

[2] Ludwig-Müller J. (2009). Plant defence—What can we learn from clubroots?. Australas. Plant Pathol..

[3] Donald C., Porter I. (2009). Integrated control of clubroot. J. Plant Growth Regul..

[4] Ludwig-Müller J., Prinsen E., Rolfe S.A., Scholes J.D. (2009). Metabolism and plant hormone action during clubroot disease. J. Plant Growth Regul..

[5] Kageyama K., Asano T. (2009). Life cycle of *Plasmodiophora brassicae*. J. Plant Growth Regul..

[6] Ludwig-Müller J., Pieper K., Ruppel M., Cohen J.D., Epstein E., Kiddle G., Bennett R. (1999). Indole glucosinolate and auxin biosynthesis in *Arabidopsis thaliana* (L.) Heynh. glucosinolate mutants and the development of clubroot disease. Planta.

[7] Siemens J., Keller I., Sarx J., Kunz S., Schuller A., Nagel W., Schmülling T., Parniske M., Ludwig-Müller J. (2006). Transcriptome analysis of *Arabidopsis* clubroots indicate a key role for cytokinins in disease development. Mol. Plant Microbe Interact..

[8] Devos S., Vissenberg K., Verbelen J.-P., Prinsen E. (2005). Infection of Chinese cabbage by *Plasmodiophora brassicae* leads to a stimulation of plant growth: Impacts on cell wall metabolism and hormone balance. New Phytol..

[9] Grsic-Rausch S., Kobelt P., Siemens J.M., Bischoff M., Ludwig-Müller J. (2000). Expression and localization of nitrilase during symptom development of the clubroot disease in Arabidopsis. Plant Physiol..

[10] Päsold S., Siegel I., Seidel C., Ludwig-Müller J. (2010). Flavonoid accumulation in *Arabidopsis thaliana* root galls caused by the obligate biotrophic pathogen *Plasmodiophora brassicae*. Mol. Plant Pathol..

[11] Neuhaus K., Grsic-Rausch S., Sauerteig S., Ludwig-Müller J. (2000). *Arabidopsis* plants transformed with nitrilase 1 or 2 in antisense direction are delayed in clubroot development. J. Plant Physiol..

[12] Dharmasiri N., Dharmasiri S., Estelle M. (2005). The F-Box Protein TIR1 is an auxin receptor. Nature.

[13] Spartz A.K., Gray W.M. (2008). Plant hormone receptors: New perceptions. Genes Dev..

[14] Christian M., Steffens B., Schenck D., Burmester S., Böttger M., Lüthen H. (2006). How does auxin enhance cell elongation? Roles of auxin-binding proteins and potassium channels in growth control. Plant Biol..

[15] Petroski M.D., Deshaies R.J. (2005). Function and regulation of cullin-RING ubiquitin ligases. Nat. Rev. Mol. Cell Biol..

[16] Santner A., Estelle M. (2009). Recent advances and emerging trends in plant hormone signalling. Nature.

[17] Guilfoyle T.J., Hagen G. (2007). Auxin response factors. Curr. Opin. Plant Biol..

[18] Reed J.W. (2001). Roles and activities of Aux/IAA proteins in Arabidopsis. Trends Plant Sci..

[19] Hagen G., Guilfoyle T.J. (2002). Auxin-responsive gene expression: Genes, promoters and regulatory factors. Plant Mol. Biol..

[20] Staswick P.E., Serban B., Rowe M., Tiryaki I., Maldonado M.T., Maldonado M.C., Suza W. (2005). Characterization of an Arabidopsis enzyme family that conjugates amino acids to indole-3-acetic acid. Plant Cell.

[21] Park J.-E., Park J.-Y., Kim Y.-S., Staswick P.E., Jeon J., Yun J., Kim S.-Y., Lee Y.-H., Park C.-M. (2007). GH3-mediated auxin homeostasis links growth regulation with stress adaptation response in Arabidopsis. J. Biol. Chem..

[22] Zhang Z., Li Q., Li Z., Staswick P.E., Wang M., Zhu Y., He Z. (2007). Dual regulation role of GH3.5 in salicylic acid and auxin signaling during Arabidopsis-*Pseudomonas syringae* interaction. Plant Physiol..

[23] Wang M.-Y., Liu X.-T., Chen Y., Xu X.-J., Yu B., Zhang S.-Q., Li Q., He Z.-H. (2012). *Arabidopsis* acetyl-amido synthetase GH3.5 involvement in camalexin biosynthesis through conjugation of indole-3-carboxylic acid and cysteine and upregulation of camalexin biosynthesis genes. J. Integr. Plant Biol..

[24] Staswick P.E., Tiryaki I. (2004). The oxylipin signal jasmonic acid is activated by an enzyme that conjugates it to isoleucine in Arabidopsis. Plant Cell.

[25] Thines B., Katsir L., Melotto M., Niu Y., Mandaokar A., Liu G., Nomura K., He S.Y., Howe G.A., Browse J. (2007). JAZ repressor proteins are targets of the SCF complex during jasmonate signalling. Nature.

[26] Steffens B., Lüthen H. (2000). New methods to analyse auxin-induced growth. II. The swelling reaction of protoplasts—A model system for the analysis of auxin signal transduction. Plant Growth Regul..

[27] Chen J.G., Ullah H., Young J.C., Sussman M.R., Jones A. (2001). ABP1 is required for organized cell elongation and division in Arabidopsis embryogenesis. Genes Dev..

[28] Fuchs I., Philippar K., Hedrich R. (2006). Ion channels meet auxin action. Plant Biol..

[29] Alix K., Lariagon C., Delourme R., Manzanares-Dauleux M.J. (2007). Exploiting natural genetic diversity and mutant resources of *Arabidopsis thaliana* to study the *A. thaliana*-*Plasmodiophora brassicae* interaction. Plant Breed..

[30] Devos S., Prinsen E. (2006). Plant hormones: A key in clubroot development. Commun. Agric. Appl. Biol. Sci..

[31] Winter D., Vinegar B., Nahal H., Ammar R., Wilson G.V., Provart N.J. (2007). An “Electronic Fluorescent Pictograph” Browser for exploring and analyzing large-scale biological data sets. PLoS One.

[32] Parry G., Calderon-Villalobos L.I., Prigge M., Peret B., Dharmasiri S., Itoh H., Lechner E., Gray W.M., Bennett M., Estelle M. (2009). Complex regulation of the TIR1/AFB family of auxin receptors. Proc. Natl. Acad. Sci. USA.

[33] Greenham K., Santner A., Castillejo C., Mooney S., Sairanen I., Ljung K., Estelle M. (2011). The AFB4 auxin receptor is a negative regulator of auxin signaling in seedlings. Curr. Biol..

[34] Dharmasiri N., Dharmasiri S., Weijers D., Lechner E., Yamada M., Hobbie L., Ehrismann J.S., Jürgens G., Estelle M. (2005). Plant development is regulated by a family of auxin receptor F box proteins. Dev. Cell.

[35] Devos S., Laukens K., Deckers P., van Der Straeten D., Beeckman T., Inzé D., van Onckelen H., Witters E., Prinsen E. (2006). A hormone and proteome approach to picturing the initial metabolic events during *Plasmodiophora brassicae* infection on Arabidopsis. Mol. Plant Microbe Interact..

[36] Hardke C.S., Ckurshumova W., Vidaurre D.P., Singh S.A., Stamatiou G., Tiwari S.B., Hagen G., Guilfoyle T.J., Berleth T. (2004). Overlapping and non-redundant functions of the Arabidopsis auxin response factors MONOPTEROS and NONPHOTOTROPHIC HYPOCOTYL 4. Development.

[37] Berleth T., Jürgens G. (1993). The role of the monopteros gene in organising the basal body region of the Arabidopsis embryo. Development.

[38] Okushima Y., Fukaki H., Onoda M., Theologis A., Tasaka M. (2007). ARF7 and ARF19 regulate lateral root formation via direct activation of *LBD*/*ASL* genes in *Arabidopsis*. Plant Cell.

[39] Schlereth A., Möller B., Liu W., Kientz M., Flipse J., Rademacher E.H., Schmid M., Jürgens G., Weijers D. (2010). MONOPTEROS controls embryonic root initiation by regulating a mobile transcription factor. Nature.

[40] Gutierrez L., Mongelard G., Floková K., Păcurar D.I., Novák O., Staswick P., Kowalczyk M., Păcurar M., Demailly H., Geiss G., Bellini C. (2012). Auxin controls Arabidopsis adventitious root initiation by regulating jasmonic acid homeostasis. Plant Cell.

[41] Tian C.-E., Muto H., Higuchi K., Matamura T., Tatematsu K., Koshiba T., Yamamoto K.T. (2004). Disruption and overexpression of auxin response factor 8 gene of Arabidopsis affect hypocotyl elongation and root growth habit, indicating its possible involvement in auxin homeostasis in light condition. Plant J..

[42] De Rybel B., Vassileva V., Parizot B., Demeulenaere M., Grunewald W., Audenaert D., van Camenhout J., Overvoorde P., Jansen L., Vanneste S. (2010). A novel Aux/IAA28 signaling cascade activates GATA23-dependent specification of lateral root founder cell identity. Curr. Biol..

[43] Kobelt P. (2000). Die Verbreitung von sekundären Plasmodien von *Plasmodiophora brassicae* im Wurzelgewebe von *Arabidopsis thaliana* nach immunhistologischer Markierung des plasmodialen Zytoskeletts. Ph.D. Thesis.

[44] Belin C., Megies C., Hauserova E., Lopez-Molina L. (2009). Abscisic acid represses growth of the Arabidopsis embryonic axis after germination by enhancing auxin signaling. Plant Cell.

[45] Mallory A.C., Bartel D.P., Bartel B. (2005). MicroRNA-directed regulation of Arabidopsis AUXIN RESPONSE FACTOR17 is essential for proper development and modulates expression of early auxin response genes. Plant Cell.

[46] Nakamura A., Nakajima N., Goda H., Shimada Y., Hayashi K., Nozaki H., Asami T., Yoshida S., Fujioka S. (2006). Arabidopsis Aux/IAA genes are involved in brassinosteroid-mediated growth responses in a manner dependent on organ type. Plant J..

[47] Jülke S., Ludwig-Müller J. (2010). Modulation of lipid transfer proteins alters clubroot development in *Arabidopsis thaliana*. Acta Hortic..

[48] Arabidopsis eFP browser. http://bar.utoronto.ca/.

[49] Bandurski R.S., Cohen J.D., Slovin J., Reinecke D.M., Davies P.J. (1995). Auxin Biosynthesis and Metabolism. Plant Hormones: Physiology, Biochemistry and Molecular Biology.

[50] Ludwig-Müller J. (2011). Auxin conjugates: Their role for plant development and in the evolution of land plants. J. Exp. Bot..

[51] Seidel C., Walz A., Park S., Cohen J.D., Ludwig-Müller J. (2006). Indole-3-acetic acid protein conjugates: Novel players in auxin homeostasis. Plant Biol..

[52] Siemens J., Glawischnig E., Ludwig-Müller J. (2008). Indole glucosinolates and camalexin do not influence the development of the clubroot disease in *Arabidopsis thaliana*. J. Phytopathol..

[53] Siemens J., Nagel M., Ludwig-Müller J., Sacristán M.D. (2002). The interaction of *Plasmodiophora brassicae* and *Arabidopsis thaliana*: Parameters for disease quantification and screening of mutant lines. J. Phytopathol..

[54] Wang H., Tian C., Duan J., Wu K. (2008). Research progresses on GH3s, one family of primary auxin-responsive genes. Plant Growth Regul..

[55] Jones B., Ljung K. (2011). Auxin and cytokinin regulate each other’s levels via a metabolic feedback loop. Plant Signal. Behav..

[56] Dohmann E., Kuhnle C., Schwechheimer C. (2005). Loss of the CONSTITUTIVE PHOTOMORPHOGENIC9 signalosome subunit 5 is sufficient to cause the *cop*/*det*/*fus* mutant phenotype in Arabidopsis. Plant Cell.

[57] Staswick P. (2013).

[58] Ludwig-Müller J., Jülke S., Bierfreund N.M., Decker E.L., Reski R. (2009). Moss *(Physcomitrella patens*) GH3 proteins act in auxin homeostasis. New Phytol..

[59] González-Lamothe R., El Oirdi M., Brisson N., Bouarab K. (2012). The conjugated auxin indole-3-acetic acid-aspartic acid promotes plant disease development. Plant Cell.

[60] Shi J.-H., Yang Z.-B. (2011). Is ABP1 an auxin receptor yet?. Mol. Plant.

[61] Scherer G.F.E. (2011). AUXIN-BINDING-PROTEIN1, the second auxin receptor: What is the significance of a two-receptor concept in plant signal transduction?. J. Exp. Bot..

[62] Klode M., Dahlke R.I., Sauter M., Steffens B. (2011). Expression and subcellular localization of *Arabidopsis thaliana* auxin-binding protein 1 (ABP1). J. Plant Growth Regul..

[63] Blatt M.R., Thiel G. (1994). K^+^ channels of stomatal guard cells: bimodal control of the K^+^ inward-rectifier evoked by auxin. Plant J..

[64] Philippar K., Fuchs I., Lüthen H., Hoth S., Bauer C.S., Haga K., Thiel G., Ljung K., Sandberg G., Böttger M., Becker D., Hedrich R. (1999). Auxin-induced K^+^ channel expression represents an essential step in coleoptile growth and gravitropism. Proc. Natl. Acad. Sci. USA.

[65] Fuchs I., Philippar K., Ljung K., Sandberg G., Hedrich R. (2003). Blue light regulates an auxin-induced K^+^ channel gene in the maize coleoptile. Proc. Natl. Acad. Sci. USA.

[66] Philippar K., Ivashikina N., Ache P., Christian M., Lüthen H., Palme K., Hedrich R. (2004). Auxin activates KAT1 and KAT2, two K^+^-channel genes expressed in seedlings of *Arabidopsis thaliana*. Plant J..

[67] Claussen M., Lüthen H., Blatt M., Böttger M. (1997). Auxin-induced growth and its linkage to potassium channels. Planta.

[68] Fähling M., Graf H., Siemens J. (2003). Pathotype-separation of *Plasmodiophora brassicae* by the host plant. J. Phytopathol..

[69] Jefferson R.A. (1987). Assaying chimeric genes in plants: The GUS gene fusion system. Plant Mol. Biol. Rep..

[70] The Arabidopsis Information Resource. http://www.arabidopsis.org.

