# Supplementary material for: The Clubroot Pathogen (Plasmodiophora brassicae) Influences Auxin Signaling to Regulate Auxin Homeostasis in Arabidopsis
Source: Plants (Basel). 2013 Nov 27;2(4):726–49. doi: 10.3390/plants2040726 (PMC4844388; doi:10.3390/plants2040726)
Supplement: Supplementary File 1 [file plants-02-00726-s001.pdf]

Figure S1. Gene expression patterns of selected *GH3* genes in roots according to the Arabidopsis eFP browser [31,48].

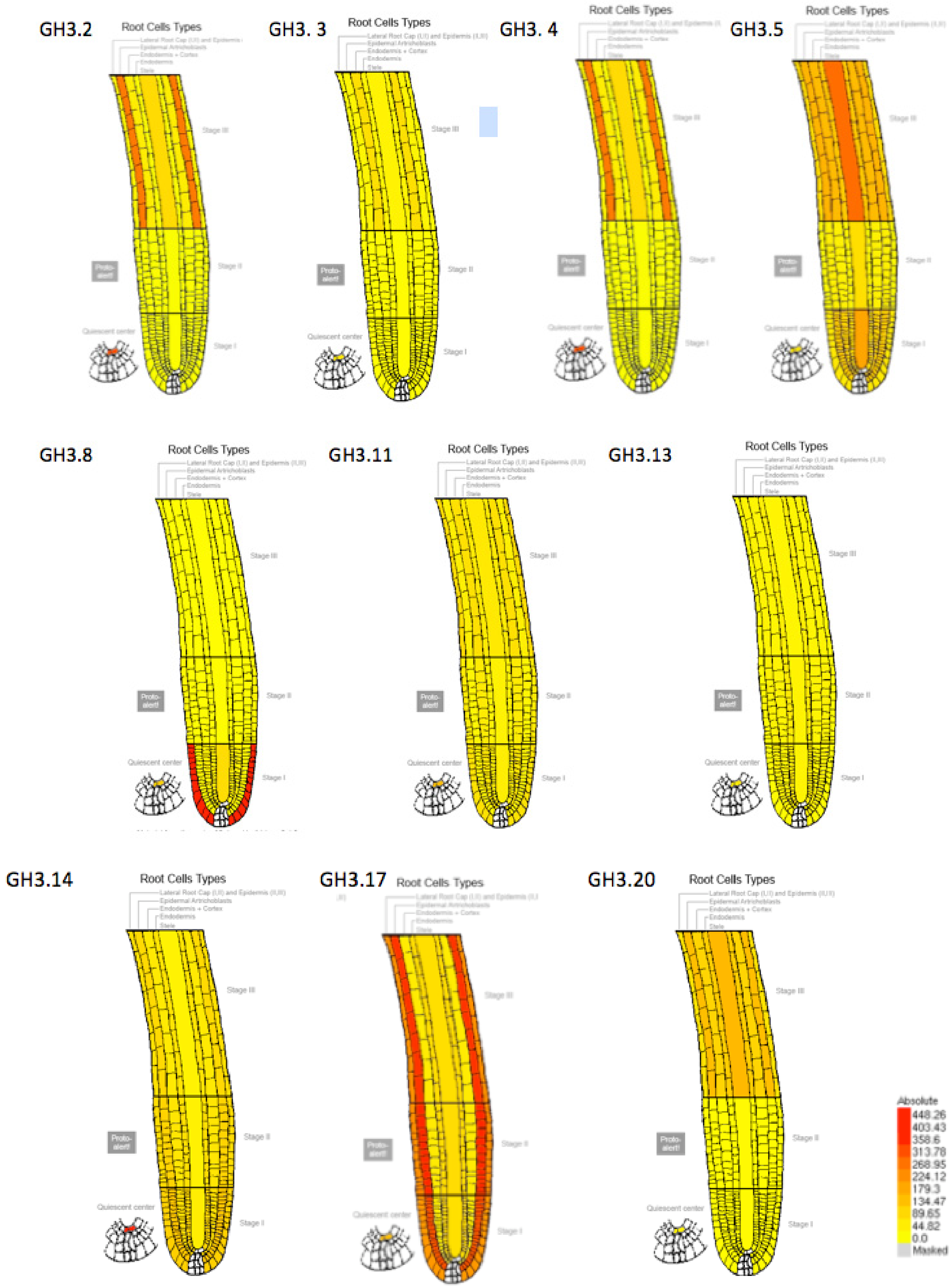

Figure S2. Shoot phenotype of Arabidopsis plants treated with the potassium channel inhibitor tetraethylammonium (TEA) at a concentration of 10 mM once a week. Treatment two times a week produced the same phenotype (data not shown). Pictures were taken of 42 day old plants.

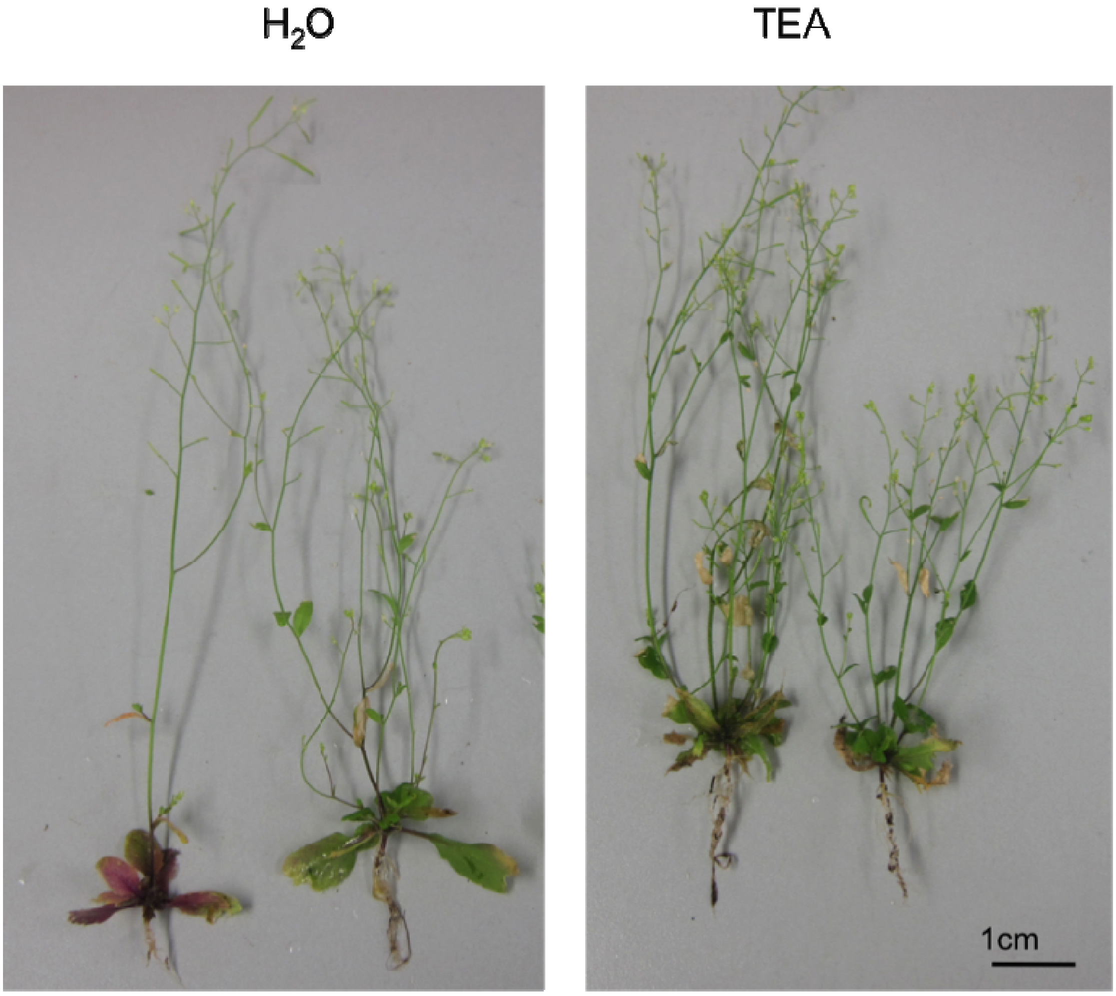

Table S1. Regulation of auxin receptor, *Aux/IAA* and *ARF* genes during clubroot compiled according to the microarray analysis (Arabidopsis ATH1; E-MEXP-254) by Siemens *et al*. [7]. Two time points were analyzed: At 10 days after inoculation (dai) (TP1) small secondary plasmodia of the pathogen are visible, but only about 20% of the host tissue was colonized with limited change of host cell and root morphology. At 23 dai (TP2) different developmental stages of the pathogen were present. More than 60% of the host root cells were colonized and the root morphology was drastically altered. In blue genes upregulated by ≥ 2-fold are indicated, in red those downregulated by ≤ 2-fold.

**Gene**	**x-fold regulation by *P. brassicae***	
	**TP1**	**TP2**
**Receptors**		
TIR1	1.24	1.87
AFB1	2.60	3.33
AFB2	−1.08	1.05
AFB3	−1.03	1.77
AFB4	1.41	1.06
AFB5	1.48	1.38
ABP1	2.06	1.85
**Transcription repressors (Aux/IAA)**		
IAA1	1.02	−1.12
IAA2	−3.57	−2.70
IAA3	−4.34	−3.33
IAA4	1.01	−1.23
IAA5	−4.00	−20.00
IAA6	−1.88	-1.11
IAA7	−1.22	5.07
IAA8	−1.28	−2.38
IAA9	−1.51	−1.02
IAA10	−2.85	−1.66
IAA11	1.16	−1.78
IAA12	1.34	−1.16
IAA13	−1.49	−2.08
IAA14	1.13	4.96
IAA15	not on array	
IAA16	−8.33	−2.70
IAA17	−1.78	−2.12
IAA18	−3.84	−2.32
IAA19	−5.00	−5.88
IAA20	1.03	2.27
IAA24	1.00	−2.50

Table S1. *Cont.*

**Gene**	**x-fold regulation by *P. brassicae***	
**Transcription repressors (Aux/IAA)**		
IAA26	−1.85	−2.32
IAA27	−3.70	−14.28
IAA28	−3.12	−3.70
IAA29	1.16	1.30
IAA30	−1.23	−1.25
IAA31	1.20	2.30
IAA32	1.76	2.53
IAA33	2.85	−1.22
IAA34	1.39	1.71
**Auxin response factors**		
ARF1	−1.96	−1.08
ARF2	−2.00	−1.05
ARF3	2.28	2.46
ARF4	1.46	−1.06
ARF5	−1.02	−2.85
ARF6	−1.26	−2.00
ARF7 (IAA21)	−1.58	−2.22
ARF8	2.10	1.53
ARF9	1.43	−1.11
ARF10	2.63	−1.11
ARF12	1.00	2.43
ARF13	1.22	−1.40
ARF14	1.01	−1.64
ARF15	−1.01	2.81
ARF16	−2.32	−1.49
ARF17	1.23	−2.27
ARF18	−1.17	−1.47
ARF19 (ARF11, IAA22)	1.31	1.37
ARF20	not on array	
ARF21/ARF22 *	1.00	2.43
ARF23	1.52	4.25

* can not be distinguished on array.

Table S2. Regulation of *GH3* gene expression during clubroot compiled according to the microarray analysis (Arabidopsis ATH1; E-MEXP-254) by Siemens *et al.* [7] and the Arabidopsis eFP browser [31,48] for induction by auxin. For description of the time points chosen see Table S1. In blue genes upregulated by ≥ 2-fold are indicated, in red those downregulated by ≤ −2-fold.

**GH3.x**	**x-fold regulation by *P. brassicae***		**Substrate**	**Mutant**	**Induction by auxin** [#]		
	TP1	TP2			30 min	1 h	3 h
1	2.03	1.20			4.6	4.7	10.8
2	1.32	6.39	IAA	YDK1	−1.2	−1.4	−1.1
3	91.98	137.52	IAA		11.0	43.5	41.1
4	1.30	31.29	IAA		20.9	45.4	60.1
5	−1.35	5.55	IAA, SA	WES1	2.8	7.1	13.2
6	1.60	−1.26	IAA	DFL1	1.8	4.2	6.4
7	1.33	−1.05			−1.4	1.6	1.0
8	3.05	−1.35			−1.6	−1.6	−1.1
9	1.78	−1.14	IAA		1.1	−1.6	−1.4
10	1.11	7.33		DFL2	1.0	1.3	−1.6
11	−2.00	−2.22	JA	JAR1	−1.2	−1.2	1.1
12	2.42	1.63	BA, (SA?)	GDG1	−2.5	−3.3	8.0
13	1.16	9.62			−1.2	1.3	1.3
14/15 *	3.38	10.03			1.0	−1.1	1.0
16	1.42	1.44			−1.2	1.0	1.6
17	2.47	1.62	IAA		1.0	−1.2	−1.2
18	−1.26	1.13			−1.6	−5.0	−2.0
19	1.32	−1.23			−5.0	1.1	2.2
20	1.52	−2.63			−1.1	−2.5	1.0

* can not be distinguished on array; [#] bar.utoronto.ca.

Table S3. Inducibility of *pGH3.2::GUS* and a mutated version in the auxin-response element (mpGH3.2::GUS). Coloring is indicated by ++, +, (+), --. Age of plants is given as days after inoculation (dai), also for mock-inoculated plants. 10 dai = 24 day old plants; 28 dai = 42 day old plants. The experiment was done three times on 10 plants for each line.

Dai	Condition	Plant line		
		wild type	pGH3.2::GUS	mpGH3.2::GUS
10	10 µM IAA	--	++	--
	control	--	+	--
	P. brassicae	--	+	--
28	10 µM IAA	--	++	--
	control	--	+	--
	P. brassicae	--	++	(+)

Table S4. All primer sequences used in this study and the corresponding PCR conditions. The first sequence is for the forward, the second for the reverse primer. The sizes of the cDNA and, in brackets, the genomic DNA PCR products are given. * The number of cycles sometimes varied for parallel independent experiments.

Name of gene	Accession number	Primer sequence (5' –> 3')	Size of PCR product	Annealing conditions (°C) / time (s)	Number of cycles *
ABP1	At4g02980	ACT GTT GCT GGC TCC GTA TT	298	58 °C/45 s	31–42
		TAA TAG GCG GCC GAG ATA TG	(783)		
AFB1	At4g03190	ATC TCG TTC CTG ATG GTT GG	789	58 °C/45 s	39–45
		ACA CCC TCA GTT CTC GCA GT	(981)		
AFB2	At4g03190	TAC GCA GCT GAG ATT CAT GG	895	58 °C/45 s	39–45
		AAC GGA AGA CGA CCA ATC AG	(974)		
ARF5	At1g19850	CTC TCG CCA AGA TTT TCC AG	358	57 °C/45 s	32–45
		CCT TAC GCA TCC CAC AAA CT	(546)		
ARF7	At5g20730	CAT GCC TCA GCT CAT CGT AA	521	54 °C/45 s	33
		GCG GCT AAA ACA AGA ACT CG	(599)		
ARF8	At5g37020	TCA ACA TCT GGA TTG GGT CA	300	57 °C/45 s	42
		CGG TGT CAA TGG TTG AAG TG	(579)		
ExpA17	At4g01630	TTC TCT CTA TTG GTC GCA ATG ATC	752<br>(859)	65 °C/30 s<br>45 °C/30 s	16<br>16
		GTT AAC GTT GCT CTT GAA GCT CTG			
IAA7	At3g23050	TGA TGA CTC AGC AGA AGA CCA	302	59 °C/45 s	42
		GTC CTC GTA GCT TGG CAC A	(560)		
IAA28	At5g25890	TAA AGT TCT GGT CGG GGA TG	298	51 °C/45 s	31–38
		AAC CCC CGA TTT CTT AAA CG	(654)		
GH3.2	At4g37390	GAA ATG ACT CGG AAC CCT GA	525	55 °C/30 s	30–39
		GCA GAG GAT GGC TTC GTT AG	(760)		
GH3.3	At2g23170	AGC CTT GCC TTT GTC TAA TCC	210	55 °C/30 s	30–37

		CCG GAG ATT CAA CGT ATT GC	(800)		
GH3.4	At1g59500	GGT TAA CTT CAT GGC GCA TT<br>CTC ATC CCA TCA CCG AGT TT	375<br>(703)	55 °C/30 s	30–39
GH3.5	At4g27260	TAT CTC AGG CGA CAC GAC CT<br>GAA TCC AGA GGC GAA GAC AG	546<br>(806)	55 °C/30 s	35–39
GH3.8	At5g51470	AAC GTC CCT GTG GTG ACC TA<br>CAC CAA CCT TCA CAA CAT CG	472<br>(700)	55 °C/30 s	40
GH3.10	At4g03400	CGA GAC GCT ACG AAG GAT TC<br>CAA GAA TCC TCC CTC CTT CC	365<br>(540)	53 °C/45 s	42
GH3.11	At2g46370	GGA AAC GCT ACT GAC CCT GA<br>CGT GAA CTA GAC CGT GAG CA	535<br>(748)	55 °C/30 s	27–35
GH3.13	AT5g13350	TGT CAA TCC TCT GTG CAA GC<br>CGG CTT CTT CAG TCT TCA CC	692<br>(815)	55 °C/30 s	40
GH3.14	At5g13360	GTC ATG ATC CCG CAG AGA AT<br>GCT GGA ATG CAG CTC ATG TA	674<br>(764)	55 °C/30 s	27–30
GH3.17	At1g28130	GCC GAA GTT GAT GCC TTC TA<br>ACC GCA GAG AAG CTG ACA GT	307<br>(717)	55 °C/30 s	27–30
GH3.20	At1g48690	TCA CTT TTC CTT CCC CAA TG<br>TGA CGT CAA ATG TGA AGC AAG	366<br>(567)	55 °C/30 s	35–40
LTP18370	At2g18370	GCA ACA ACA AAG AAA CCC CTA<br>TTA GGA CAA GAT GGA CCA TTG A	448<br>(885)	58 °C/30 s	30
TIR1	At3g62980	AGC TTA ACC GAG CTG TTC CA<br>GGT CAT TTG ACG GCA GAA GT	506<br>(691)	58 °C/45 s	32–40
YLS8	At5g08290	AAG AGC GTC TCG TCG TCA TT<br>CGG CTT CCA AAT TTC ATT TC	572<br>(1037)	54 °C/45 s	27
PbActin	AY452179	AGC TGG CGT ACG TGG CGC AG<br>CCT TGA CGC GCA TCG ACG AC	342<br>(342)	62 °C/40 s	30
